# Analysis of Age-Dependent Transcriptomic Changes in Response to Intracerebral Hemorrhage in Mice

**DOI:** 10.3389/fnmol.2022.908683

**Published:** 2022-05-23

**Authors:** Xinhui Li, Wensong Yang, Yiqing Shen, Fangyu Liu, Xin Xiong, Qingyuan Wu, Zhongsong Xiao, Xun Yang, Ruozhi Dang, Anatol Manaenko, Peng Xie, Qi Li

**Affiliations:** ^1^Department of Neurology, The First Affiliated Hospital of Chongqing Medical University, Chongqing, China; ^2^NHC Key Laboratory of Diagnosis and Treatment on Brain Functional Diseases, The First Affiliated Hospital of Chongqing Medical University, Chongqing, China; ^3^Department of Neurology, Chongqing Hospital of Traditional Chinese Medicine, Chongqing, China; ^4^Department of Neurology, Chongqing University Three Gorges Hospital, Chongqing, China

**Keywords:** aging, inflammation, intracerebral hemorrhage, type I interferon, transcriptomics, neurological deficit

## Abstract

Age is a well-known risk factor that is independently associated with poor outcomes after intracerebral hemorrhage (ICH). However, the interrelationship between age and poor outcomes after ICH is not well defined. In this study, we aimed to investigate this relationship based on collagenase-induced ICH mice models. After being assessed neurological deficit 24 h after ICH, mice were euthanized and brain perihematomal tissues were used for RNA-sequencing (RNA-seq). And then the functions of differentially expressed genes (DEGs) identified by RNA-seq were analyzed using Gene Ontology (GO) analysis, Kyoto Encyclopedia of Genes and Genomes (KEGG) analysis, Ingenuity Pathway Analysis (IPA) and protein-protein interaction (PPI) analysis. In addition, we performed real-time quantitative polymerase chain reaction (RT-qPCR) for validation of candidate DEGs. In the behavioral tests, aged mice presented significantly worse neurological function than young mice and greater weight loss than aged sham controls 24 h after ICH. In DEGs analysis, ICH affected the expression of more genes in young mice (2,337 DEGs) compared with aged mice (2,005 DEGs). We found aged mice exhibited increased brain inflammatory responses compared with young animals and ICH induced significant activation of the interferon-β (IFN-β) and IFN signaling pathways exclusively in aged mice. Moreover, further analysis demonstrated that ICH resulted in the activation of cytosolic DNA-sensing pathway with the production of downstream molecule type I IFN, and the response to type I IFN was more significant in aged mice than in young mice. In agreement with the results of RNA-seq, RT-qPCR indicated that the expression of candidate genes of cyclic GMP-AMP synthase (cGAS), Z-DNA-binding protein 1 (ZBP1), and IFN-β was significantly altered in aged mice after ICH. Taken together, our study indicated that compared to young animals, aged mice exhibit increased vulnerability to ICH and that the differences in transcriptional response patterns to ICH between young and aged mice. We believe that these findings will facilitate our understanding of ICH pathology and help to translate the results of preclinical studies into a clinical setting.

## Introduction

Intracerebral hemorrhage (ICH) is a cerebrovascular disease with high mortality (Schrag and Kirshner, 2020). Although ICH accounts for only 10–20% of stroke cases, it has a 30-day mortality rate of up to 50% and a high morbidity rate, making the disease a major health problem in our increasingly aging population (An et al., 2017; Cordonnier et al., 2018).

Aging describes physiological progressive functional decline at the molecular and systemic levels. Age has a significant effect on internal immunoinflammatory responses to acute injury and might induce chronic brain dysfunction (Sinha et al., 2014; Bilkei-Gorzo et al., 2017). The previous studies reported that age results in impaired immunity, including the loss of naive T cells and the accumulation of NK cells (Smithey et al., 2018; Jin et al., 2021). In acute brain injury after neurological disorder, such as ICH, subarachnoid hemorrhage and ischemic stroke, active immune cells and secreted proinflammatory factors comprise a coordinated and comprehensive network (Xue and Yong, 2020). In response to injury signals, brain intrinsic active neuroglia and vascular endothelial cells recruit peripheral circulating monocytes and neutrophils accelerating blood-brain barrier disruption as well as exacerbating brain edema (Askenase et al., 2021). A recent study suggested that the age-related increase in peripheral CCL12 expression plays an important role in aggravating brain injury in an experimental ICH model (Huang et al., 2020). However, the details of the age-related response to ICH in the brain are still unclear.

In recent years, numerous drugs have been shown to be efficacious in preclinical models of ICH, but the great majority of them have failed in clinical trials (Selim et al., 2018; Bai et al., 2020; Ren et al., 2020). This may be because most of the preclinical data were acquired in young rodent models of ICH. However, patient age is an important factor that significantly affects outcomes after ICH (Pasi et al., 2021). Therefore, we hypothesized that the senescence-associated changes that occur in the brain after ICH can be more precisely modeled in old animals than in young ones. In clinical studies, an increasing ICH incidence rate was identified in patients above 75 years old (Lioutas et al., 2020). Moreover, senescence-associated pathologic alterations were recently observed in studies on ICH (Wang et al., 2019; Kinoshita et al., 2020). For example, a study on haptoglobin gene knockout mice demonstrated that some interesting changes were displayed in young groups exclusively, including less striatal microgliosis, thalamic astrogliosis and brain damage at later timepoint (Leclerc et al., 2019). However, few researchers have explored the effect of age on the development of brain injury after ICH. Thus, clarifying the mechanisms underlying the development of more severe senescence-associated brain injury following ICH is of profound therapeutic significance.

RNA sequencing (RNA-seq) is a useful tool for functional and global transcriptomic analysis. Recently, the expression of monocyte-related genes in swine models within the first 6 h after ICH was analyzed by RNA-seq (Walsh et al., 2019). Evidence from RNA-seq analysis demonstrated the cross-talk between specific monocyte subsets and vascular smooth muscle cells contributes to the detrimental pathological changes after ICH (Winkler et al., 2022). In another, RNA-seq revealed that myelin and oligodendroglia-related genes showed the most robust alterations in the aged mouse brain (Rivera et al., 2021). However, few animal experiments have employed aged animal models for RNA-seq in ICH-related studies. Therefore, we aimed to investigate the relationship between aging and ICH at the gene level by RNA-seq.

## Materials and Methods

### Animals

Aged (20–22 months, *n* = 18) and young (8–10 weeks, *n* = 17) male C57BL/6 mice were obtained from the Animal Experimental Center of Chongqing Medical University. The mice were housed in a specific pathogen-free environment at a constant temperature (23 ± 2°C) and under appropriate lighting conditions (12-h light-dark cycle). The animals had free access to food and water. Institutional ethics committee approval was obtained for the present study. All experimental procedures complied with the National Institutes of Health Guide for the Care and Use of Laboratory Animals. All efforts were made to minimize the suffering of the mice. One young animal and two aged animals died and were excluded from the study. The animals were divided into four groups: the young sham, young ICH, aged sham, and aged ICH groups (eight animals per group).

### Intracerebral Hemorrhage Model

**FIGURE 1:**
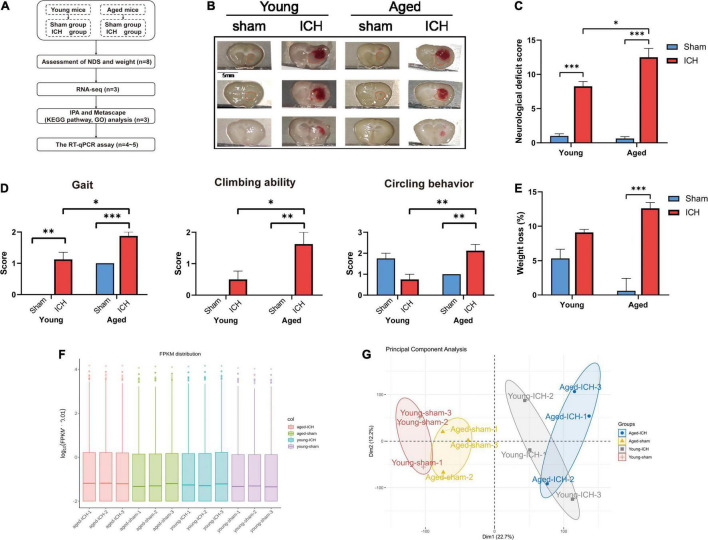
Experimental protocols, sampling regions, the results of behavioral tests, quantification and quality control of RNA sequencing data. **(A)** Experimental flowchart. ICH, intracerebral hemorrhage; NDS, neurological deficit score; RNA-seq, RNA-sequencing; IPA, ingenuity pathway analysis; KEGG, Kyoto Encyclopedia of Genes and Genomes; GO, gene ontology; RT-qPCR, real-time quantitative polymerase chain reaction. **(B)** Representative macrographs of coronal brain sections and sampling regions (marked with red traces) from the four groups. **(C)** Compared to sham surgery, ICH induced significant neurological dysfunction in both aged and young animals (*n* = 8); Compared to young mice, ICH induced more severe neurological deficits in aged mice. **(D)** The comparison of gait, climbing ability, and circling behavior scores between aged and young mice. **(E)** Body weight was significantly decreased in aged mice with ICH compared with aged sham mice (*n* = 8). **(F)** The box plot of fragments per kilobase of transcript sequence per millions mapped reads (FPKM) distribution per sample. **(G)** The principal component analysis plot of mRNA expression in the four groups (*n* = 3). Dim, dimension. The behavioral data were shown as the mean ± SEM. *P < 0.05, **P < 0.01, and ***P < 0.001.

The experimental flowchart is shown in Figure 1A. In this study, a model of collagenase-induced ICH was employed (Bobinger et al., 2020). The mice were anesthetized by inhalation of 4–5% isoflurane, and anesthesia was maintained with 1.5–2% isoflurane. An incision was made in the scalp to expose bregma. Then, the mouse was placed in the prone position, and its head was fixed in a stereotaxic frame (Rivard, Shenzhen, China). ICH was induced by infusion of sterile collagenase IV (Sigma, St. Louis, MO, United States). A small burr hole was created (from bregma: 0.6 mm anterior and 2.3 mm to the right), and a Hamilton needle (Hamilton, Bonaduz, Switzerland) was inserted to a depth of 3.7 mm. Then, 0.075 U of collagenase (dissolved in 0.4 μl of sterile saline) was infused into the right basal ganglia at a rate of 0.5 μl/min using a microinfusion pump (Rivard, Shenzhen, China). The needle was left in place for 5 min before being retracted at a rate of 1 mm/min. After the needle was retracted, the incision was sutured, and the mice were allowed to recover. Sham-operated mice underwent all surgical procedures, but received injection of 0.4 μl sterile saline. The body weight of each animal was measured before and 24 h after the operation.

### Behavioral Tests

Twenty-four hours after ICH, the neurological deficits of the mice were assessed by the neurological deficit score (NDS) using seven subtests that evaluated body symmetry, gait, climbing ability, circling behavior, forelimb symmetry, compulsive circling behavior, and whisker reactivity (Clark et al., 1997). Performance on all subtests was scored on a scale of 0–4, with a maximum score of 28, indicating the most severe neurological dysfunction.

### Sampling

Anesthetized mice were perfused intracardially with 10–20 ml 4°C phosphate-buffered saline. The whole brains were immediately extracted and cut into 5–6 equally spaced coronal sections (1 mm thick). Representative macrographs of coronal brain sections from each group and the regions of sampling are shown in Figure 1B. Perihematomal tissues in ipsilateral caudate putamen were carefully dissected and collected from slices in which the hematoma was visible on an ice-cold plate and snap-frozen in liquid nitrogen. Corresponding tissues were harvested from sham control slices and frozen in the same way. Finally, all samples were stored in a −80°C freezer until use.

### Transcriptomics

Perihematomal tissues from two or three mice were pooled as one biological sample to reduce individual differences; thus, there were three biological replicates per group (Conesa et al., 2016; Gong et al., 2019). Three micrograms of RNA from each sample was used for transcriptomic analysis. The RNA concentration was quantified using a Qubit^®^ RNA Assay Kit with a Qubit^®^ 2.0 Fluorometer (Life technologies, Carlsbad, CA, United States), and RNA integrity was analyzed on an Agilent Bioanalyzer 2100 system (Agilent Technologies, Santa Clara, Calif, CA, United States). The paired-end sequencing library was constructed using the NEBNext^®^ Ultra™ RNA Library Prep Kit for Illumina^®^ (NEB, Beverly, MA, United States), and each sample was assigned an index code. Next, the RNA was reverse transcribed into cDNA, and cDNA fragments with a length of 250–300 bp were screened using the AMPure XP system (Beckman Coulter, Beverly, MA, United States). The cDNA was then amplified by polymerase chain reaction (PCR), and the PCR products were subsequently purified on the AMPure XP system. The sequencing library was assessed using the Agilent Bioanalyzer 2100 system and then clustered accordingly. The library was loaded in an Illumina platform sequencer for 125/150 bp paired-end sequencing.

Raw data were processed into clean reads after deleting reads of low-quality and reads with ploy-*N* or adapter using in-house perl scripts. Then clean paired-end reads were matched to the reference genome based on gene annotation data from Ensembl database (version: GRCm38.p6, Mus Musculus.GCF_000001635.26) using Hisat2 (v2.0.5). Expression levels of genes were quantified through FeatureCounts v1.5.0-p3 and presented as FPKM (Fragments Per Kilobase of transcript sequence per Millions mapped reads). A principal component analysis (PCA) algorithm was used, and the differences in mRNA expression among groups were assessed. DEGs were identified by DESeq2 R package (v1.16.1), which analyzed data according to negative binomial distribution. Fold change (FC) > 1.5 and <0.67 (the reciprocal of 1.5) with a *P*-value < 0.05 were used as the thresholds for identifying upregulated and downregulated differentially expressed genes (DEGs) between selected groups according to previous studies (Den Low et al., 2019; Wang et al., 2020).

### Bioinformatics Analysis

For identification of overlapping DEGs between different comparisons, a Venn diagram tool was used.^1^ To identify the genes that showed an age-ICH interaction, we identified two additional gene sets that were significantly affected by ICH in the aged group (Table 1). In the two gene sets, the FC of each gene in the aged ICH group (vs. aged sham controls) was at least 1.5 times that of the young ICH group (vs. young sham controls) or less than 0.67 times that in the young ICH group (vs. young sham controls). Volcano plots and heatmaps showing the DEGs were presented. The subsequent functional enrichment analysis was based on DEGs. Omics data were uploaded to the Metascape database (Zhou et al., 2019)^2^ for Gene Ontology (GO) analysis and Kyoto Encyclopedia of Genes and Genomes (KEGG) pathway analysis and uploaded to the Ingenuity Pathway Analysis (IPA) software^3^ (IPA^®^, Qiagen, Redwood City, CA, United States) for pathway enrichment. The significantly enriched pathways and GO terms were identified by *P* < 0.01. A z-score > 2 was consider as the criterion of the activated pathway in IPA analysis. Search Tool for the Retrieval of Interacting Genes/Proteins (STRING) database 11.5^4^ were used for protein-protein interaction (PPI) analysis. Confidence score was set as >0.4 to identify the DEGs for constructing PPI networks, which were visualized in Cytoscope software 3.8.2^5^ (Shannon et al., 2003). Then Molecular Complex Detection (MCODE) and CytoNCA plug-in were performed to access clustered modules and degree centrality, respectively^6^ (Bader and Hogue, 2003). In module analysis, the parameters were set as a degree cutoff of 2, node score cutoff of 0.2, k-core of 2 and maximum depth of 100, and the functions of the nodes from modules were investigated in DAVID database 6.8^7^ (Dennis et al., 2003).

**TABLE 1:** The two additional gene sets used for enrichment analysis.

Number	Gene sets	The criterion
1	“More upregulated in the aged ICH group”	(1) FC (aged ICH vs. sham) > 1.5, *P* < 0.05 (2) log_2_FC (aged ICH vs. sham) − log_2_FC (young ICH vs. sham log_2_FC) > log_2_1.5
2	“More downregulated in the aged ICH group”	(1) FC (aged ICH vs. sham) < 0.67, *P* < 0.05 (2) log_2_FC (aged ICH vs. sham) − log_2_FC (young ICH vs. sham) < log_2_0.67

### Real-Time Quantitative Polymerase Chain Reaction

Total mRNA was extracted from each sample (young sham, *n* = 5; young ICH, *n* = 4; aged sham, *n* = 4; aged ICH, *n* = 4) with TRIzol (Invitrogen, Carlsbad, CA, United States). For mRNA transcription, the PrimeScript^®^ RT Reagent Kit (RR047A, Takara, Shiga, Japan) and a Bio-Rad T100 thermal cycler (Bio-Rad, United States) were used. RT-qPCR was performed by using SYBR^®^ Premix Ex Taq™ II (RR820A, Takara, Shiga, Japan) on a Light Cycler 96 system (Roche, Mannheim, Germany). The reactions were performed in a volume of 20 μL following the manufacturer’s instructions. Glyceraldehyde 3-phosphate dehydrogenase (GAPDH) was chosen as an internal control. The comparative CT method (2^–ΔΔCT^) was used to analyse the data. The sequences of the primers (Sangon Biotech, Shanghai, China) used for RT-qPCR are shown in Table 2.

**TABLE 2:** Primers used for real-time quantitative polymerase chain reaction (RT-qPCR).

Gene		Primers (5′–3′)
GAPDH	Forward	AGGTCGGTGTGAACGGATTTG
	Reverse	TGTAGACCATGTAGTTGAGGTCA
cGAS	Forward	GAGGCGCGGAAAGTCGTAA
	Reverse	TTGTCCGGTTCCTTCCTGGA
STING	Forward	GGTCACCGCTCCAAATATGTAG
	Reverse	CAGTAGTCCAAGTTCGTGCGA
TBK1	Forward	ACTGGTGATCTCTATGCTGTCA
	Reverse	TTCTGGAAGTCCATACGCATTG
IRF3	Forward	GAGAGCCGAACGAGGTTCAG
	Reverse	CTTCCAGGTTGACACGTCCG
IRF7	Forward	GAGACTGGCTATTGGGGGAG
	Reverse	GACCGAAATGCTTCCAGGG
ZBP1	Forward	AAGAGTCCCCTGCGATTATTTG
	Reverse	TCTGGATGGCGTTTGAATTGG
IFN-α	Forward	AAGCCATCCCTGTCCTGAGTGAG
	Reverse	TTGTATTCCATGCAGCAGATGAGTCC
IFN-β	Forward	CTGGGTGGAATGAGACTATTGT
	Reverse	AAGTTCCTGAAGATCTCTGCTC

### Statistical Analysis of the Behavioral and Polymerase Chain Reaction Results

All statistical analyses were performed using the commercially available software SPSS 23.0 (IBM, Armonk, NY, United States). The data are expressed as the mean ± SEM. First, the normality of data from four groups and differences in variance between groups were checked. For comparisons of two groups, independent-sample *t*-test was used to analyse normally distributed data and the Mann–Whitney *U*-test was used for nonparametric analysis. For comparisons of multiple groups, one-way ANOVA following by the Bonferroni *post hoc* test was used to analyse normally distributed data, and the Kruskal-Wallis test followed by Dunn’s multiple comparisons test was used for nonparametric analysis. The level of significance was set at *P* < 0.05.

## Results

### Intracerebral Hemorrhage Induced More Severe Neurological Dysfunction in Aged Mice Than in Young Mice

The neurological functions of the animals were evaluated by NDS. Significant neurological dysfunction was observed in both aged and young mice with ICH compared to their respective sham controls (Figure 1C; *P* < 0.001). Post-ICH neurological deficits were significantly more severe in aged mice than in young mice (Figure 1C; *P* < 0.05). After ICH, the scores of the aged mice in the gait, climbing ability, and circling behavior subtests were significantly worse than those of young mice (Figure 1D; *P* < 0.05). Before surgery, the weight of the aged mice was significantly higher than that of the young mice (*P* < 0.001). Aged mice with ICH (*P* < 0.001), but not young mice with ICH (*P* > 0.05), presented more significant weight loss than their respective sham controls, and there was no significant difference in weight loss between aged mice and young mice after ICH (Figure 1E; *P* > 0.05).

### Both Aging and Intracerebral Hemorrhage Induced Changes in mRNA Expression

**FIGURE 2:**
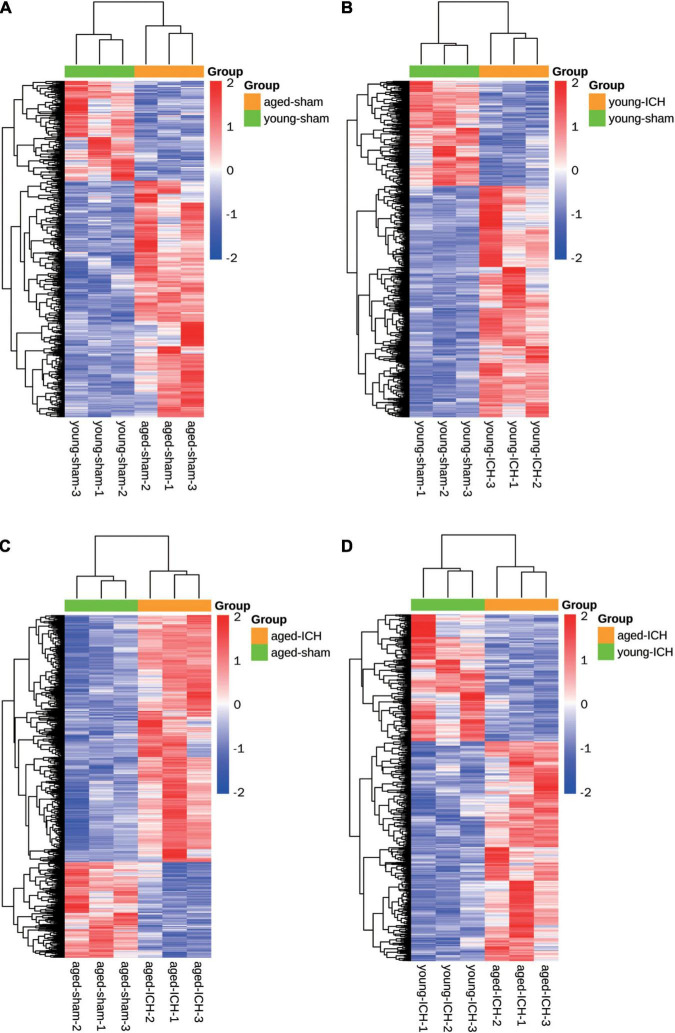
Heatmaps of different pairwise comparisons. The criterion for identification DEGs is FC > 1.5 or FC < 0.67, *P* < 0.05. **(A)** Young sham vs. aged sham. **(B)** Young ICH vs. young sham. **(C)** Aged ICH vs aged sham. **(D)** Aged ICH vs. Young ICH. ICH, intracerebral hemorrhage.

We acquired more than 85 million of clean sequence reads in average among 12 samples and the sequencing depth, quality and the rates of alignment were presented in Sheet 1 of Supplementary Table 1. The box plot presented the distribution of mRNA expression levels by FPKM among four groups (Figure 1F). As demonstrated in the PCA plot (Figure 1G), the mRNA profiles of the four groups, i.e., the young sham, young ICH, aged sham, and aged ICH groups, were different. Complete coordinate information for the 12 samples in the PCA plot is shown in Sheet 2 of Supplementary Table 1. The mRNA profiles of ICH group were different compared with respective sham controls in young/aged mice, and gene expression patterns were different between young mice and aged mice in sham/ICH groups. The above results ensured the reliability of subsequent bioinformatics analysis. To gain insight into the variation in expression profiles of DEGs between groups, we constructed heatmaps by unsupervised hierarchical clustering analysis (Figure 2). We found that the samples from the four groups clustered together upon selected pairwise comparisons. The above RNA-seq data suggested that aged mice have different mRNA profiles than young mice and that the DEG data were credible.

### Differentially Expressed Genes in the Brain Between Aged and Young Mice and Related Functional Analysis

**FIGURE 3:**
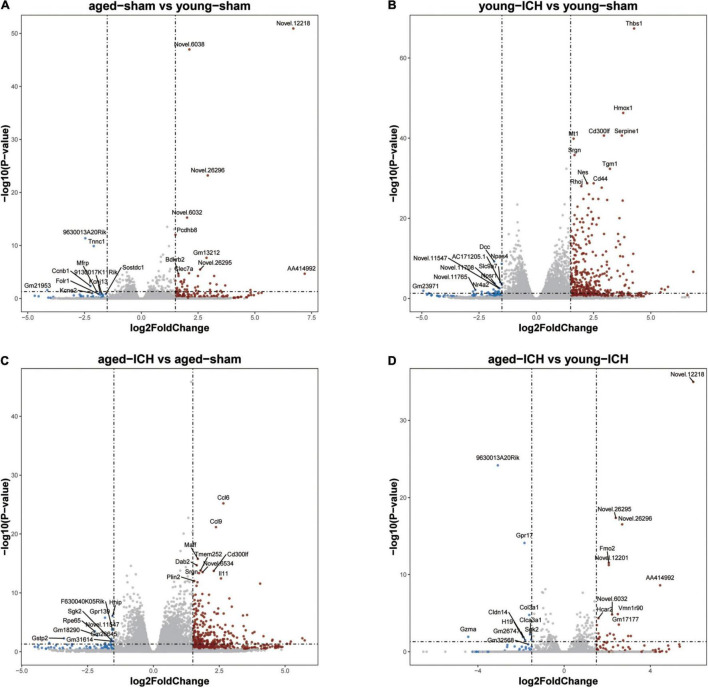
Volcano plots of selected pairwise comparisons. The criterion for identification DEGs is FC > 1.5 or FC < 0.67, *P* < 0.05. **(A)** Young sham vs. aged sham. **(B)** Young ICH vs. young sham. **(C)** Aged ICH vs. aged sham. **(D)** Aged ICH vs. Young ICH. ICH, intracerebral hemorrhage.

**FIGURE 4:**
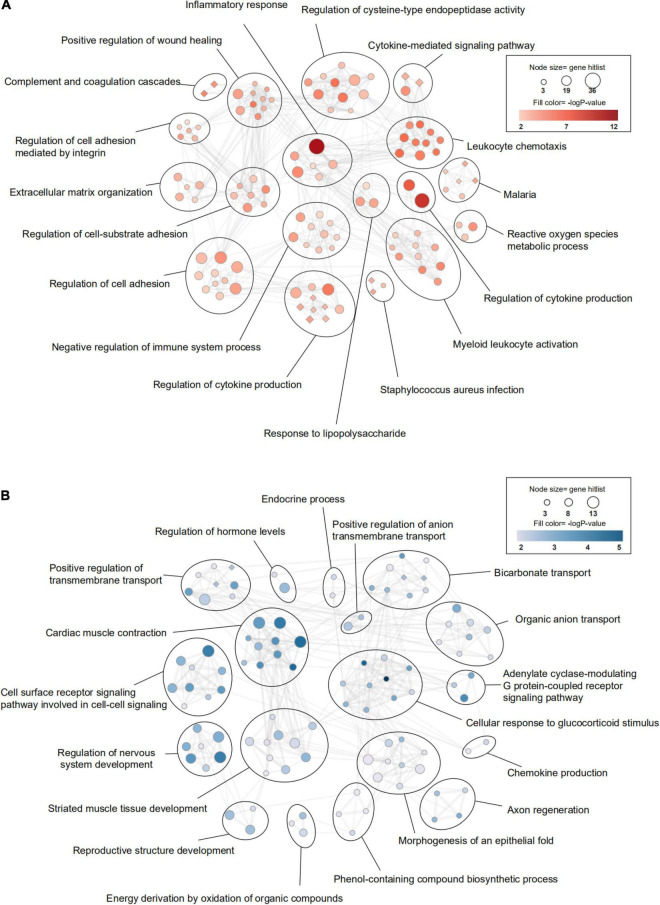
Transcriptomic differences in the brain between young and aged mice. **(A)** Gene Ontology (GO) and KEGG (Kyoto Encyclopedia of Genes and Genomes) enrichment analysis of significantly upregulated differentially expressed genes (DEGs) and **(B)** downregulated DEGs between young mice and aged mice after sham surgery was performed using Metascape. The significantly enriched GO terms and pathways (*P* < 0.01, z-score > 2) are presented in enrichment maps, and all of them are grouped into clusters based on membership similarities. The color of the nodes in the maps indicates the *P* value, and the size of the nodes reflects the number of genes in the hit list. Terms with a similarity > 0.3 are connected by lines. Circular nodes reflect GO terms, and diamond-shaped nodes reflect KEGG pathways. *n* = 3 per group.

Firstly, we investigated the effect of aging alone on mice by comparing gene expression in aged and young mice after sham surgery. We identified 641 DEGs, which included 451 upregulated genes and 190 downregulated genes, in aged sham mice compared with young sham mice (Figures 2A, 3A). As shown in Figure 4A and Sheet 1 of Supplementary Table 2, functional enrichment analysis of the upregulated genes revealed that the inflammatory response was increased in the brains of aged mice compared with the brains of young mice. Identification of the functional clusters of leukocyte chemotaxis, regulation of cytokine production, and regulation of cell adhesion showed that significant infiltration and activation of peripheral leukocytes occurred in the brains of aged mice. In addition, detection of clusters associated with the activation of reactive oxygen species metabolism suggested an increase in oxidative stress reactions. On the other hand, functional enrichment analysis of the downregulated genes in aged mice compared with young mice revealed disruption of axons and signal transduction between cells (Figure 4B and Sheet 2 of Supplementary Table 2).

### Comparison of Intracerebral Hemorrhage Induced Differentially Expressed Genes Between Aged and Young Mice and Functional Enrichment Analysis

**FIGURE 5:**
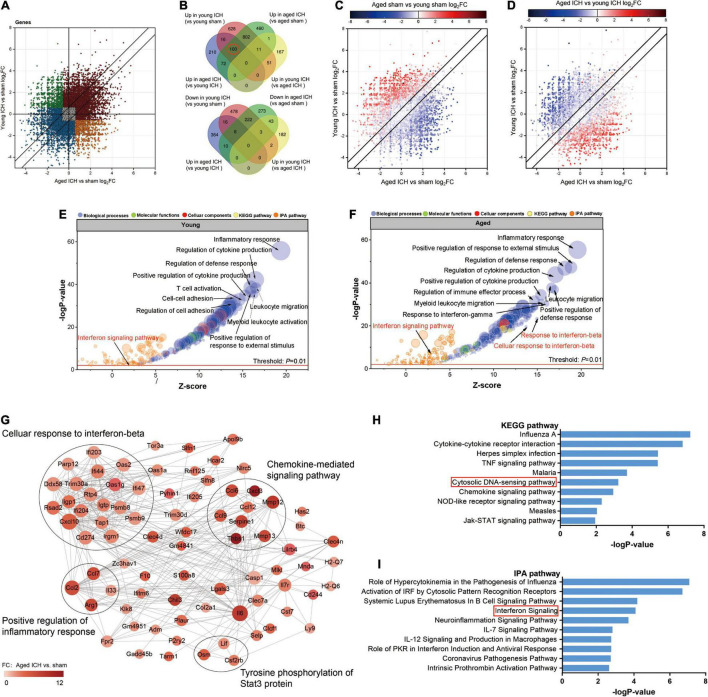
Comparison of differentially expressed genes (DEGs) and functional clusters between young and aged mice with intracerebral hemorrhage (ICH). **(A)** Scatterplot showing the log_2_ foldchange (FC) of genes between young and aged mice after ICH. Genes with an FC > 1.5 or FC < 0.67 are colored. **(B)** The Venn diagram showing overlap of up- or downregulated DEGs between selected pairwise groups. **(C)** Scatterplots of the log_2_FC of genes between young and aged mice after ICH. The color of the scatters indicates the log_2_FC of the genes in aged mice vs. young mice after sham surgery **(D)** or after ICH. **(E)** Bubble plots comparing ICH-induced alteration in functional clusters from several databases (including Metascape website and Ingenuity pathway analysis (IPA) software) in young mice **(F)** and aged mice. The Gene Ontology (GO) terms included biological process (BP), cellular component (CC) and molecular function (MF) terms. The size of the bubbles reflects the number of genes in each term, and the color of bubbles reflects the enriched category. **(G)** Protein-protein interaction (PPI) analysis and **(H)** Kyoto Encyclopedia of Genes and Genomes (KEGG) pathway enrichment analysis of 100 overlapping genes in the Venn diagram (marked with a red circle), overlapping genes among “upregulated in the young ICH group vs. the young sham group”, “upregulated in the aged ICH group vs. the aged sham group” and “upregulated in the aged ICH group vs. the young ICH group”. In the PPI network, the size of the nodes reflects the degree and the color reflects the FC value of aged ICH vs. sham. The most significant BP in GO enrichment analysis was marked in every module. **(I)** Pathways in the IPA system associated with genes that showed stronger upregulation after ICH in aged mice (“more upregulated in the aged ICH group”). *n* = 3 per group.

Next, we investigated the effect of ICH alone by comparing gene expression between animals with ICH and their age-matched controls. A large number of DEGs were identified in young mice (2,337 genes in total, 1,608 upregulated and 729 downregulated genes) and aged mice (2,005 genes in total, 1,446 upregulated and 559 downregulated genes) compared with their sham controls (Figures 2B,C, 3B,C). In addition, there were 628 DEGs (190 upregulated and 438 downregulated genes) between ICH groups of two ages (Figures 2D, 3D). As shown in Figure 5A, there were more upregulated genes than downregulated genes, and the overall FC range of the upregulated genes was greater than that of the downregulated genes. To further explore the overlapping DEGs between young and aged mice after ICH, we generated a Venn diagram. There were a large number of overlapping genes between young and aged mice (Figure 5B), Interestingly, the overlapping DEGs between aged ICH vs. aged sham and aged ICH vs. young ICH were more than that between young ICH vs. young sham and young ICH vs. aged ICH (Figure 5B), which indicated the expression of DEGs affected by age and ICH in aged mice were more in number compared with young ones. We then explored the genes that were upregulated to a greater extent in young mice after ICH (log_2_FC of young ICH vs. sham > the log_2_FC of aged ICH vs. sham), of which most also showed elevated expression in aged sham mice vs. young sham mice (Figure 5C). However, the FC value of these genes in young mice after ICH were not greater than those in aged mice after ICH (Figure 5D).

Most of the GO terms enriched for the DEGs in the young ICH group vs. the young sham group and the aged ICH group vs. the aged sham group were similar and were mainly involved in the biological process (BP) of immune and inflammatory response (Figures 5E,F and Sheets 3, 4 of Supplementary Table 2), such as inflammatory response, regulation of defense response and positive regulation of response to external stimulus. ICH-induced DEGs also enriched in immune cells responses including leukocyte migration, myeloid leukocyte activation, proliferation and differentiation of monocytes, and the migration and chemotaxis process of neutrophils and monocytes in both young and aged mice (Figures 5E,F and Sheets 3, 4 of Supplementary Table 2). Surprisingly, we found that the BPs of response to interferon-β (IFN-β) and cellular response to IFN-β were enriched exclusively in aged mice (Figures 5E,F and Sheets 3, 4 of Supplementary Table 2; −log *P* = 23.30 and −log *P* = 19.56, respectively). Moreover, IPA predicted that the IFN signaling pathway was activated in aged mice after ICH but not in young mice after ICH (Figures 5E,F and Sheets 1, 2 of Supplementary Table 3; z-score > 2 in aged mice and z-score < 2 in young mice).

### Functional Analysis of Differentially Expressed Genes Showing a More Robust Changes in Aged Mice After Intracerebral Hemorrhage

To evaluate the effect of aging and ICH on mice, we next explored the upregulated genes marked in a red circle in Figure 5B (100 in total) that showed the most robust changes in aged mice compared with young mice by PPI analysis and KEGG enrichment analysis. The module with the most nodes and highest score was involved in the BP of cellular response to IFN-β (Figure 5G and Sheet 1 of Supplementary Table 4), which ranked the top one in the enrichment result (Sheet 2 of Supplementary Table 4). Interestingly, we also found that ICH significantly activated the cytosolic DNA-sensing pathway in aged mice (Figure 5H and Sheet 3 of Supplementary Table 3; −log *P* = 3.23). The downstream products of this pathway are type I IFN (IFN-α, IFN-β, etc.) and proinflammatory cytokines (IL-1β, IL-6, NF-kb, etc.).

**FIGURE 6:**
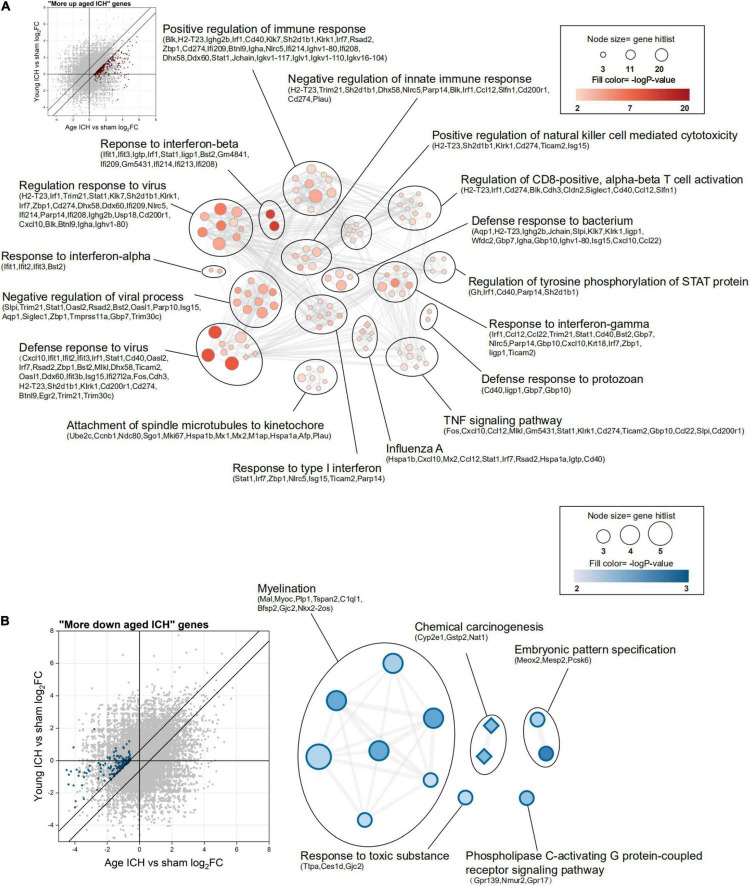
Functional annotation of differentially expressed genes (DEGs) between young and aged mice after intracerebral hemorrhage (ICH). **(A)** The scatterplot shows DEGs that were more strongly upregulated after ICH in aged mice (“more upregulated in the aged ICH group”), and the enrichment map shows significantly enriched Gene Ontology (GO) terms (circles) and Kyoto Encyclopedia of Genes and Genomes (KEGG) pathways (diamonds) for these genes (*P* < 0.01, z-score > 2). **(B)** The scatterplot and enrichment map for DEGs but for DEGs that were more strongly downregulated after ICH in aged mice (“more downregulated in the aged ICH group”). *n* = 3 per group.

Then, we sought to analyse potentially harmful genes that exhibit greater upregulation after ICH in aged mice (“more upregulated in the aged ICH group”, 258 genes in total). IPA analysis of these 258 genes showed that the IFN signaling pathway was activated (Figure 5I and Sheet 4 of Supplementary Table 3). As shown in Figure 6A and Sheet 5 of Supplementary Table 2, the enrichment map included two KEGG pathway clusters, namely, the Toll-like receptor signaling pathway and tumor necrosis factor signaling pathway. According to GO enrichment analysis of the genes in the map, the response to IFN-β was the most significantly enriched term (−log *P* = 15.28), demonstrating that genes associated with IFN-β were activated by ICH much more strongly in aged mice than in young mice. According to the functional clusters, responses to type I IFN and IFN-α, as well as the regulation of tyrosine phosphorylation of signal transducer and activator of transcription (STAT) protein, were also significantly activated. In addition, GO terms associated with the regulation of CD8 positive, alpha-beta T cell activation, positive regulation of natural killer cell-mediated cytotoxicity, and other inflammatory responses were enriched. Some of the GO terms were immune-related BPs, such as response to IFN-γ, cellular response to lipopolysaccharide, and regulation of response to biotic stimulus. The attachment of spindle microtubules to kinetochore was also enriched. Consistent with the above results, the most significant BP in the larger module of PPI network was the defense response to virus. A distinct finding is that DEGs in the smaller module were related to cell cycle, which including Mki67, Ccnb1, Ndc80, Ube2c, and E2f8 (Supplementary Figure 2A and Sheets 3, 4 of Supplementary Table 4).

In contrast, there were 143 genes exhibiting greater downregulation in aged mice after ICH than in young mice after ICH (“more downregulated in the aged ICH group”). As shown in Figure 6B and Sheet 6 of Supplementary Table 2, these genes are involved in myelination, chemical carcinogenesis, embryonic pattern specification, response to a toxic substance, and phospholipase C-activating G protein-coupled receptor signaling pathway. The enrichment result of the 2 modules of PPI network were similar with the results (Supplementary Figure 2B and Sheets 5, 6 of Supplementary Table 4).

### Validation of Candidate Genes

**FIGURE 7:**
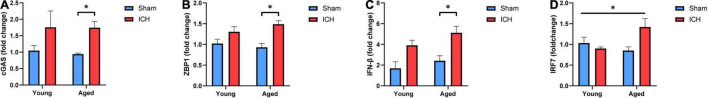
Validation of candidate genes. **(A)** The mRNA expression levels of Cyclic GMP-AMP synthase (cGAS), **(B)** Z-DNA-binding protein 1 (ZBP1), **(C)** Interferon-β (IFN-β) and **(D)** interferon regulatory factor 7 (IRF7) were measured by real-time quantitative polymerase chain reaction (RT-qPCR). The mRNA expression is reported as the FC vs. young mice after sham surgery. The data are shown as the mean 2^–ΔΔCT^ value ± SEM; young sham: *n* = 5; young ICH: *n* = 4; aged sham: *n* = 4; aged ICH: *n* = 4; *P < 0.05.

Finally, we performed qPCR to validate the changes in the expression of several genes in the cytosolic DNA-sensing pathway among the groups. As shown in Figures 7A–D, the mRNA expression of cyclic GMP-AMP synthase (cGAS), Z-DNA-binding protein 1 (ZBP1), and IFN-β was significantly increased in aged mice with ICH compared with their sham controls (*P* < 0.05). This increase was not observed in young mice with ICH compared with their sham controls (*P* > 0.05). There were no significant differences in the expression of these genes between young mice and aged mice after either sham surgery or ICH. Additionally, multiple comparisons showed a significant difference in IRF7 expression level among groups (*P* < 0.05), but there was no difference in pairwise comparisons, and there were no significant differences in the expression of stimulator of interferon genes (STING), TANK binding kinase (TBK1), interferon regulatory factor 3 (IRF3), or IFN-α among the four groups (*P* > 0.05; Supplementary Figures 1A–D). Taken together, these findings suggested that ICH induced the upregulation of cGAS, ZBP1, and IFN-β expression in aged mice.

## Discussion

In this study, we explored the effects of aging on ICH-induced brain injury in a murine model. We found that ICH induced worse behavioral performances in aged mice than in young mice, leading to more severe neurological deficits and body weight loss. We also found that the responses to ICH at the gene level were highly similar between young and aged mice but varied in magnitude. Finally, we postulated that the worse outcome after ICH in aged mice was associated with the response to type I IFN.

Our present study demonstrated that, in accordance with previous findings, neurological dysfunction was more severe in aged mice than in young mice 24 h after ICH (Gong et al., 2004). In the gait, climbing ability, and circling behavior subtests, aged mice with ICH performed worse than young mice with ICH. This indicated that ICH-induced weakness of the forelimbs was more prominent in aged mice than in young mice. Moreover, our experiments revealed significantly greater weight loss in aged mice with ICH than in their sham controls. We did not observe this effect in young mice. Based on these findings, we hypothesize that ICH-induced injury may affect the ability to consume food using the forelimbs and result in temporal anorexia in elderly animals. Therefore, we determined that the combination of aging and ICH caused worse neurological impairment and significant weight loss in mice on the first day after ICH.

Numerous studies have indicated that brain inflammation increases with age in both mice and humans (Soysal et al., 2016; Thevaranjan et al., 2017; Denver et al., 2018). Others have reported that age induces alterations in synaptic structure and neurons (Petralia et al., 2014; Navakkode et al., 2018). These findings are in line with our study, which showed that the expression of a large number of proinflammatory genes was upregulated and that the expression of genes related to axonal integrity and cell–cell signal transduction was downregulated between the aged sham group and the young sham group. These changes may be associated with microglia-mediated prolonged and excessive inflammation in the aging brain, which results in neuronal death and synaptic damage (Griciuc et al., 2013; Jonsson et al., 2013). In addition, we hypothesize that an abnormal increase in the number of oligodendrocytes and astrocytes in the aging brain is also responsible for these changes (Spitzer et al., 2019; Gollihue and Norris, 2020). Collectively, these and other findings indicate that age sensitizes the brain to more severe injury after ICH.

We then explored how the brains of young and aged mice respond to ICH. We demonstrated that a large number of DEGs in the brains of young and aged mice compared with their sham controls were involved in the immune inflammatory response after ICH. Previous preclinical research has confirmed the essential role of inflammation in brain exposed to ICH using the integrative multi-omics technique at gene and protein levels and it is worthy of noting that competing endogenous RNA (ceRNA) network based on the whole RNA-seq data implied lncRNA and microRNA may be involved in molecular mechanism of brain injury after ICH (Cao et al., 2020). In our results, ICH-induced immune cell response occured in perihematomal tissues including chemotaxis and migration of monocytes, neutrophils and leukocytes in both ages groups. The present results are in line with others report that indicated monocytes from peripheral circulation and mobilized leukocytes from immune organ were the key components of secondary brain injury after ICH (Bai et al., 2020). In addition, previous analysis of mice perihematomal tissue has reported that the recruitment of neutrophils in acute phase of ICH via NK cells amplifies regional brain neuroinflammation and exacerbates neurological function deficits (Li Z. et al., 2020). Of interest, we found DEGs of “more upregulated in the aged ICH group” enriched in the GO term of attachment of spindle microtubules to kinetochore, indicating that proinflammatory cell proliferation may be increased in aged mice after ICH.

Notably, the response to IFN-β was activated exclusively in aged mice after ICH. Interestingly, genes that showed more stronger upregulation in aged mice after ICH were enriched for the response to IFN-β, IFN-α, and type I IFN. We next focused on the functions of overlapping genes that exhibited stronger upregulation in the brains of aged mice after ICH, which were identified by a Venn diagram. PPI analysis identified cellular response to IFN-β was the most significant BP based on DEGs from the largest module. Subsequent KEGG pathway analysis indicated an upstream pathway related to type I IFN production, namely, the cytosolic DNA-sensing pathway. In this pathway, cGAS, a key upstream sensor, is activated by binding to double-stranded DNA (dsDNA) and produces the second messenger cGAMP (Sun et al., 2013). At the endoplasmic reticulum, STING detects cGAMP, translocates to the Golgi apparatus, and is phosphorylated by TBK1. Upon binding to phosphorylated STING, activated IRF3/7 subsequently translocates to the nucleus and triggers the production of proinflammatory cytokines and type I IFN (Zhang et al., 2012). ZBP1 can also detect and bind to dsDNA and enhance its association with IRF3 and TBK1 (Takaoka et al., 2007; Ponnusamy et al., 2021).

The cytosolic DNA-sensing pathway can be activated not only by foreign agents, such as viruses and DNA (Liu et al., 2015) but also by self-DNA, i.e., nuclear, mitochondrial, or extracellular DNA. Accumulation of DNA might be the result of senescence, neurodegeneration, or brain injury after ischemic stroke (Jauhari et al., 2020; Li Q. et al., 2020). In our study, we hypothesized that the cumulative effect of aging and ICH led to the production of dsDNA, possibly derived from dead neurons, glia and other cells, and that dsDNA was probably able to activate this pathway. Abnormal regulation of this pathway has adverse effects, including acceleration of disease progression, neurodegeneration, and neuroinflammation (Paul et al., 2021). The validation results demonstrated that the mRNA expression of cGAS, ZBP1, and IFN-β was increased in aged mice (*P* < 0.05) but not in young mice (*P* > 0.05) after ICH and that the *P-*value of the difference in IRF7 mRNA expression in multiple comparisons was less than 0.05. However, it is worth noting that there was a tendency for the levels of IRF7 to be increased in the ICH group compared with the sham group, especially in aged mice. Taken together, these findings suggested that ICH induced upregulation of cGAS and ZBP1 expression in aged mice. Upregulation of the expression of these genes may be associated with overproduction of IFN-β, which we presume is one of the major factors leading to more severe brain injury after ICH in aged mice. This is in agreement with the effects of IFN-β previously observed in models of traumatic brain injury (Barrett et al., 2020). The current study reveals a novel approach for promoting innate immunity after ICH that takes into consideration age-dependent differences in the brain’s capacity to respond to ICH at the gene level.

Cyclic GMP-AMP synthase is involved in defense against antiviral infection (Jeffries and Marriott, 2020). Inhibition of cGAS can attenuate neuroinflammation after ischemic stroke in mice (Li Q. et al., 2020; Liao et al., 2020). Notably, cGAS also mediates cellular senescence (Yang et al., 2017). ZBP1 not only acts as an essential intermediate hub in the cytosolic DNA-sensing pathway mentioned above but has also been reported to be required for IFN-β-induced necroptosis *in vitro* (Yang et al., 2020). IFN-β, an important member of the type I IFN family, acts downstream of cGAS in this pathway. It is a detrimental factor that aggravates injury in the aged brain after ICH. Mounting evidence has indicated type I IFN magnify cellular and molecular immune response in age-related neurological disorder and pathological changes. Transcriptomics analysis of aged subjects observed exclusive phenotype microglia expressed type I IFN-related genes in human and mice brain (Deczkowska et al., 2017; Olah et al., 2018). A recent RNA-seq analysis between young and aged traumatic brain injury mice reported age-related upregulation of cGAS and type I IFN, which appears to be closely related with microglia-mediated neuroinflammation (Barrett et al., 2021). Except for microglia, astrocytes are the other key players in response to type I IFN according to a proteomics study (Viengkhou et al., 2021). Of interest, previous studies have reported that tPA-induced ICH and traumatic brain injury alter the type I IFN response and STING-mediated type I IFN response, respectively, in mice, thus exacerbating neuroinflammation (Abdullah et al., 2018; Wang et al., 2021). Furthermore, type I IFN is involved in accelerating neurodegenerative disease progression and regulating microglial phenotype, as previously observed in chronic neurodegeneration (Nazmi et al., 2019). Based on these findings, we hypothesized that cGAS, ZBP1, and IFN-β are crucially involved in the development of brain injury after ICH. However, in a multiple sclerosis model, activation of the STING-dependent type I IFN response by the antiviral drug ganciclovir reduces neuroinflammation and microglial reactivity (Mathur et al., 2017). These inconsistencies may be caused by differences in the pathology of the different diseases.

In microglia, astrocytes, and neurons, type I IFN binds to the heterodimeric receptor and activates Janus kinase/STAT (Ivashkiv and Donlin, 2014). STAT1, STAT2, and IRF9 form a nuclear complex that triggers the transcription of proinflammatory cytokines (IL-1β, IL-6, NF-kb, etc.) (Hu et al., 2021). Excessive type I IFN signaling activates microglia and astrocytes, leading to the massive release of proinflammatory cytokines. This in turn results in damage to the blood-brain barrier, infiltration of neutrophils, and neurodegeneration (Hui and Herrup, 2015; Sochocka et al., 2017). Unfortunately, we did not find differences in the mRNA expression of STING, IRF3, IFN-α, and TBK1 among the four groups (*P* > 0.05) in our validation assay, possibly due to the limited sample size used for RT-qPCR (*n* = 4–5). Based on our findings and a literature review, we assume that the cGAS-STING pathway mediates the type I IFN response and that the response plays a crucial role in age-associated injury after ICH.

Excepted for the immune-related responses, we also found cell cycle as an important BP in the functional enrichment results based on gene set of “more upregulated in the aged ICH group”, including Fos, Mki67, Sgol1, Rrm2, Rps27a, Ccnb1, Ndc80, Ube2c, and E2f8. A recent transcriptomic analysis study demonstrated cell cycle plays a crucial role in the spinal cord injury of aged mice (Hao et al., 2018). The drug involving cell cycle, such as β3-adrenergic innervation, has been reported to be applied in ICH experiment, which was considered as a potential target for ICH treatment (Shi et al., 2021). Among the above nine genes, latest studies have reported that Rrm2 could regulated cGAS-STING signaling pathway and the expression of IFN-β (Jiang et al., 2021), and Rps27a is one of new nuclear partners of STING, with the function of modulating dsDNA-triggered innate immune responses and binding the interactive factors of IRF3 transcript (Dixon et al., 2021). On the other hand, enrichment analysis of the more strongly downregulated genes in aged mice after ICH revealed that the largest functional cluster was myelination. This is in agreement with a recent RNA-seq experiment using the brains of aged and young mice. The analysis indicated that myelin- and oligodendroglia-related genes were among the most altered (Rivera et al., 2021). These results show that the combination of aging and ICH alter the expression of myelin-related genes and indicate the therapeutic importance of targeting white matter injury in ICH patients.

The current study has several potential limitations. The first is the relatively small sample size. The second limitation is that we only considered a single time point, i.e., 24 h after ICH. Although the selected time point is representative of the acute phase of brain injury in ICH, further efforts are needed to fully elucidate the injury mechanism using a larger sample size and studying changes in subsequent phases of injury is necessary. Overall, our data analysis revealed age-related changes under the influence of ICH based on RNA-seq. However, performing further manipulation of gain or loss of function is necessary for achieving solid evidence on effective regulating molecules related to pathological mechanism of brain injury after ICH.

## Conclusion

In conclusion, our study provides new insight into the different transcriptional responses to ICH between young and aged mice, which will improve our understanding of ICH pathology and help to translate the results of preclinical studies to a clinical setting.

## Data Availability Statement

The raw RNA sequencing data can be found in the NCBI with accession GEO number GSE200575.

## Ethics Statement

The animal study was reviewed and approved by the Ethical Committee of Chongqing Medical University.

## Author Contributions

QL, PX, WY, and XL conceived and designed the study. XL, WY, QW, YS, FL, ZX, XY, XX, and RD performed the experiment. XL, WY, and YS performed data collection. XL wrote the manuscript. QL, AM, and WY revised the manuscript. All authors analyzed the data, reviewed the final manuscript, and approved the submitted version.

## Conflict of Interest

The authors declare that the research was conducted in the absence of any commercial or financial relationships that could be construed as a potential conflict of interest.

## Publisher’s Note

All claims expressed in this article are solely those of the authors and do not necessarily represent those of their affiliated organizations, or those of the publisher, the editors and the reviewers. Any product that may be evaluated in this article, or claim that may be made by its manufacturer, is not guaranteed or endorsed by the publisher.

## Footnotes

1
http://bioinformatics.psb.ugent.be/webtools/Venn/

2
https://metascape.org

3
https://www.qiagenbioinformatics.com

4
https://string-db.org

5
https://cytoscape.org/

6
https://apps.cytoscape.org/apps/

7
https://david.ncifcrf.gov/summary.jsp

## Supplementary Material

Supplementary Figure 1 **(A)** The mRNA expression levels of stimulator of interferon genes (STING), **(B)** TANK binding kinase (TBK1), **(C)** interferon regulatory factor 3 (IRF3) and **(D)** interferon-α (IFN-α) were measured by real-time quantitative polymerase chain reaction (RT-qPCR). The mRNA expression is reported as the FC vs. young mice after sham surgery. The data are shown as the mean 2^–ΔΔCT^ value ± SEM; young sham: *n* = 5; young ICH: *n* = 4; aged sham: *n* = 4; aged ICH: *n* = 4; ns means *P* > 0.05.

Supplementary Figure 2 Protein-protein interaction network analysis for differentially expressed genes (DEGs) of **(A)** “more upregulated in the aged intracerebral hemorrhage (ICH) group” and **(B)** “more down regulated in the aged ICH group”.

## Abbreviations

ICH: intracerebral hemorrhage
DEGs: differentially expressed genes
RNA-seq: RNA-sequencing
RT-qPCR: real-time quantitative polymerase chain reaction
IFN-β: interferon-β
NDS: neurological deficit score
FC: fold change
GO: gene ontology
KEGG: Kyoto Encyclopedia of Genes and Genomes
IPA: ingenuity pathway analysis
PCA: principal component analysis
PC: principal component
GAPDH: glyceraldehyde 3-phosphate dehydrogenase
BP: biological process
MF: molecular function
CC: cellular component
STAT: signal transducer and activator of transcription
cGAS: cyclic GMP-AMP synthase
ZBP1: Z-DNA -binding protein 1
IRF3/7: interferon regulatory factor 3/7
STING: stimulator of interferon genes
TBK1: TANK binding kinase
PPI: protein–protein interaction
STRING: search tool for the retrieval of interacting genes/proteins
MCODE: molecular complex detection
FDR: false discovery rate
FPKM: fragments per kilobase of transcript sequence per Millions mapped reads
ceRNA: competing endogenous RNA.
