# Supplementary figures and images for: Analysis of Age-Dependent Transcriptomic Changes in Response to Intracerebral Hemorrhage in Mice
Source: Front Mol Neurosci. 2022 May 23;15:908683. doi: 10.3389/fnmol.2022.908683 (PMC9169040; doi:10.3389/fnmol.2022.908683)

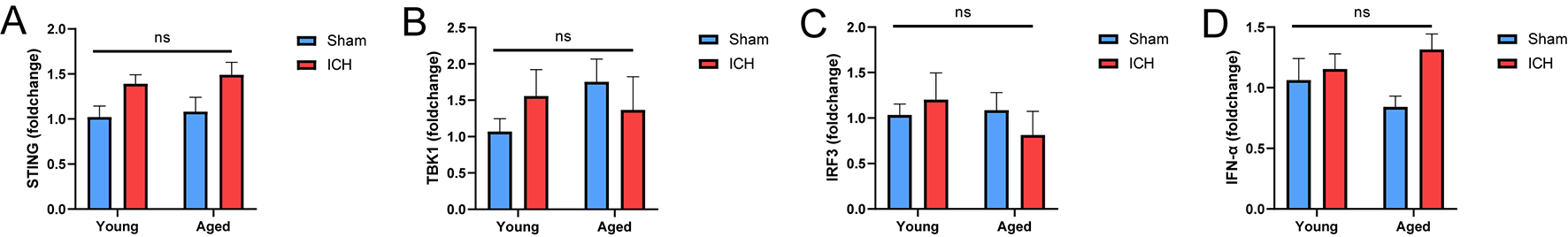

Supplement: Supplementary Figure 1 — (A) The mRNA expression levels of stimulator of interferon genes (STING), (B) TANK binding kinase (TBK1), (C) interferon regulatory factor 3 (IRF3) and (D) interferon-α (IFN-α) were measured by real-time quantitative polymerase chain reaction (RT-qPCR). The mRNA expression is reported as the FC vs. young mice after sham surgery. The data are shown as the mean 2–ΔΔCT value ± SEM; young sham: n = 5; young ICH: n = 4; aged sham: n = 4; aged ICH: n = 4; ns means P > 0.05. [file Image_1.TIF]

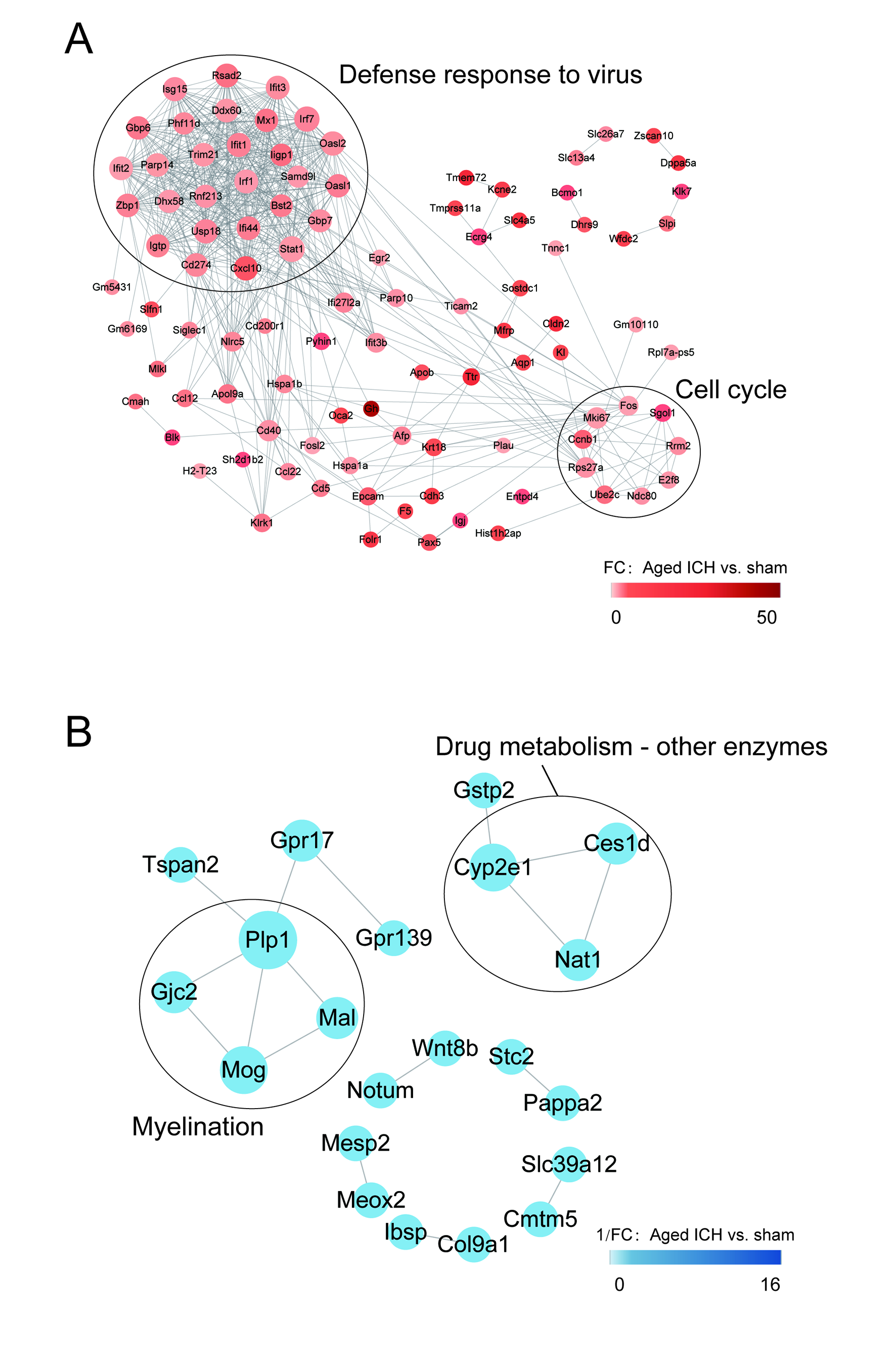

Supplement: Supplementary Figure 2 — Protein-protein interaction network analysis for differentially expressed genes (DEGs) of (A) “more upregulated in the aged intracerebral hemorrhage (ICH) group” and (B) “more down regulated in the aged ICH group”. [file Image_2.tif]
